# Diseases of Women

**Published:** 1879-11

**Authors:** 


DISEASES OF WOMEN. By Dawson Tait, G. R. C. S., surgeon to the Bermington Hospital, for women, and consulting surgeon (for diseases of women) to the West Brunswick Hospital, etc., etc. Second edition, thoroughly revised and enlarged, specially prepared for "Wood's Library," Wm. Wood & Co., New York. 1879.

The arrangement of this work is somewhet new, and yet compendious and complete. The general subject matter is interspersed with individual ideas of the author, and is altogether interesting and instructive. In this day of special practice, floods of books upon the various specialties are poured upon us. Out of this great bulk we are permitted to meet, at times, something practically valuable, or commendable for its scientific and clinical accuracy and sincerity. We are impressed with the belief that the work before us is of this character, and congratulate Messrs. Wood & Co. upon their happy selection.

